# Machine Learning Approaches to Early Detection of Parkinson’s Disease Using Speech Analysis Technique

**DOI:** 10.3390/neurolint18050088

**Published:** 2026-05-10

**Authors:** Mohammad Amran Hossain, Enea Traini, Francesco Amenta

**Affiliations:** 1 Telemedicine and Telepharmacy Centre, School of Medicinal and Health Products Sciences, University of Camerino, 62032 Camerino, Italy; enea.traini@unicam.it; 2 Research Department, International Radio Medical Centre (C.I.R.M.), 00144 Rome, Italy; presidenza@cirm.it

**Keywords:** Parkinson’s disease (PD), machine learning (ML), voice, speech, speaker diarization, spontaneous dialogue

## Abstract

Background: Parkinson’s disease (PD) is a progressive neurodegenerative disorder that affects millions globally, particularly those in the elderly population. Several occupational exposures typical of maritime environments are recognized or suspected risk factors for PD, warranting attention within occupational health frameworks. The disease is characterized by motor symptoms such as tremor, rigidity, and bradykinesia, as well as non-motor impairments including speech abnormalities. Objective: Early diagnosis is crucial for effective disease management but remains challenging due to symptoms overlapping with normal aging and other neurological conditions. This study presents a machine learning (ML)-based approach for the early diagnosis of PD using speech signal analysis. Methods: We employed six supervised ML classifiers to differentiate between PD patients and healthy controls based on vocal features. The experimental dataset, MDVR-KCL, consists of speech recordings from both reading tasks and spontaneous dialogs, collected via mobile devices. From these recordings, we extracted Mel-Frequency Cepstral Coefficients (MFCCs), Gammatone Frequency Cepstral Coefficients (GTCCs), and acoustic features such as jitter, shimmer, and harmonic-to-noise ratio. These features capture a broad range of prosodic, spectral, and articulatory characteristics associated with PD-related speech impairments. Speaker diarization was applied in spontaneous dialog recordings to separate participant speech. Hyperparameter tuning was performed using GridSearchCV with 10-fold cross-validation, while final model evaluation was conducted using Leave-One-Subject-Out Cross-Validation (LOSOCV) to ensure subject-independent performance assessment. Results: In the read-text task, the SVM model performed exceptionally, yielding 95.45% accuracy, 94.62% sensitivity, 95.97% specificity, an F1-score of 94.12%, and an AUC of 0.98 with an MCC value of 0.90, for GTCCs with the acoustic features. In the spontaneous dialog task, the XGB model demonstrated the highest overall performance across all metrics, with a test accuracy of 83.7%, a sensitivity of 76.3.9%, a specificity of 88.9%, an F1-score of 79.5%, an AUC value of 0.88, and an MCC value of 0.66. Conclusions: Comparable results were obtained on both spontaneous dialog and reading speech subsets, demonstrating the robustness of the approach across different speaking contexts. These results demonstrate the effectiveness of integrating cepstral and acoustic features with machine learning models for non-invasive PD classification. The findings support the use of speech-based digital biomarkers in early PD detection and highlight the potential for developing scalable tools. This work highlights the potential of speech-based digital diagnostics to support clinical decision-making and improve patient outcomes.

## 1. Introduction

Parkinson’s disease (PD) is a progressive neurodegenerative disorder that affects millions of people worldwide [1]. It primarily results from the gradual loss of dopaminergic neurons in the substantia nigra, a brain region essential for motor control. This degeneration leads to motor symptoms such as bradykinesia (slowness of movement), muscle rigidity, and resting tremors [2]. A significant challenge to early detection is that these symptoms often manifest only after 50–60% of the dopaminergic neurons have degenerated [3] and 60–80% of their striatal projections have degenerated [4].

PD mainly affects older adults over the age of 50, with approximately 10% of cases classified as early-onset, with symptoms beginning before age 40 [5]. Several occupational exposures typical of maritime environments are recognized or suspected risk factors for PD, warranting attention within occupational health frameworks. The disease often starts with mild symptoms, such as tremors or stiffness in one limb, and gradually progresses to include speech impairment, cognitive decline, and behavioral changes. In advanced stages, dementia may also occur [6]. These clinical features often overlap with normal aging, which complicates early and accurate diagnosis [5].

The global burden of PD has significantly increased in recent decades, with cases rising from 2.5 million in 1996 to 8.5 million in 2019 [7]. Factors contributing to this rise include aging populations, improved diagnostic capabilities, and environmental influences [2]. Conventional diagnostic procedures frequently rely on invasive and empirically based clinical evaluations [8]. These approaches are typically expensive, technically demanding, and lack the sensitivity required for early-stage detection. Therefore, there is a growing need for alternative diagnostic methods that are accurate, cost-effective, non-invasive, and scalable.

Speech analysis has emerged as a promising non-invasive technique for detecting PD [9]. Voice changes, such as hypokinetic dysarthria, a motor speech disorder marked by reduced pitch variation, phonatory instability, and articulation deficits, are among the early signs of the disease [2]. These changes can be measured through various acoustic features, including jitter, shimmer, formant frequencies, voice onset time, and vowel space area [10,11].

Mel-Frequency Cepstral Coefficients (MFCCs) are widely used in speech processing to capture the spectral characteristics of the vocal tract. They effectively model the timbre and articulation patterns that are often altered in PD speech. Gammatone Frequency Cepstral Coefficients (GTCCs), which simulate the cochlear filtering properties of human hearing, are also useful for identifying speech impairments in PD due to their sensitivity to vocal instability and reduced intensity.

Machine learning (ML) and deep learning (DL) approaches have shown strong performance in analyzing speech signals for PD detection. These methods can learn complex patterns in vocal features and distinguish between healthy individuals and those with PD. This study investigates the use of ML and DL models trained on MFCCs, GTCCs, and acoustic features from both reading tasks and spontaneous dialog to develop accurate, scalable tools for automatic early PD detection and monitoring.

### Related Work

During our literature review and background study, we identified six studies that previously used the MDVR-KCL [12] dataset for PD detection. In this section, we focus exclusively on these works as relevant prior studies. Below, we briefly summarize the objectives and methodologies adopted in each study.

Yousif et al. [13] developed a multimodal framework that combined speech and handwriting data. From the speech recordings, they extracted acoustic features and converted them into spectrogram images to train and test the convolutional neural networks (CNNs). Additionally, they used numerical features from voice recordings to train traditional classifiers such as Naïve Bayes (NB), Support Vector Machine (SVM), Decision trees (DT), and K-Nearest Neighbors (KNN). Their findings indicated that combining image-based and numerical features improved classification performance.

Di Cesare et al. [14] employed speaker diarization to process spontaneous dialog tasks, segmenting the audio stream into homogeneous sections based on speaker identity. They extracted MFCCs and GTCCs from audio signals and used these features to train ML models. The study evaluated the performance of three algorithms: Cubic-SVM, Fine-KNN, and a Wide Neural Network (WNN) on MFCCs, GTCCs, and a combination of both feature sets for PD classification.

Reddy and Akku [15] address the challenge of cross-linguistic generalization in PD detection using exemplar-based sparse representations. They analyzed speech recordings from English and Spanish speakers and extracted low-level descriptors (LLDs), including MFCCs, line spectral pair frequencies, and pitch-based features like jitter and shimmer. Their methodology involved two sparse coding techniques: L1-Regularized Least Squares (L1LS) and Non-Negative Least Squares (NNLS). These sparse representations helped identify discriminative patterns relevant to PD across languages.

Klempíř et al. [16] evaluated the use of self-supervised speech representations for PD detection. Specifically, they used wav2vec 2.0 embeddings on three datasets, including recordings of rhythmically repeated syllables and speech from English and Italian speakers. They compared these embeddings with MFCC features in both classification and regression tasks. Their results showed that wav2vec-based features capture complex speech characteristics indicative of PD more effectively than MFCCs alone.

Rohit et al. [17] proposed a hybrid decision support system for PD detection based on acoustic features extracted from the MDVR-KCL dataset. They computed 17 features, including pitch, jitter, harmonics-to-noise ratio, and shimmer. Feature selection techniques were applied to identify the most significant features. Classification was performed using KNN, XGBoost, Random Forest, and Logistic Regression. Their study highlighted the importance of ensemble methods and feature selection in enhancing model performance.

Huang et al. [18] constructed both a decision tree and a residual neural network models to distinguish PD patients from healthy individuals using speech data. They extracted 12 acoustic features from the audio signals, including harmonic-to-noise ratio, peak change, and periodic change. Their approach demonstrated the utility of combining deep and classical models for robust voice-based PD classification.

These studies collectively demonstrate the versatility of the MDVR-KCL dataset and the diverse range of techniques that can be employed for PD detection through speech analysis. From sparse representations and self-supervised learning to hybrid ML pipelines and multimodal approaches, the existing literature provides a solid foundation for further research in this domain.

## 2. Materials and Methods

### 2.1. Dataset

In this study, we employed the publicly accessible Mobile Device Voice Recordings at King’s College London (MDVR-KCL) dataset [12], which contains speech recordings from both healthy controls and patients diagnosed with PD, representing a range of disease severities, including early and advanced stages. The recordings were acquired using the “Toggle Recording App” installed on a Moto G4 smartphone, resulting in high-quality audio files with a sampling rate of 44.1 kHz and a bit depth of 16 bits. The raw, uncompressed recordings were stored directly onto the device’s external SD card in the standard WAVE file format (.wav).

Participants were instructed to perform two speech tasks. In the first task, everyone read aloud the standardized paragraph *“The North Wind and the Sun.”* Depending on their cognitive and linguistic capacity, some participants were also asked to read an additional technical excerpt titled *“Tech. Engin. Computer applications in geography snippet”.* The second task involved a spontaneous dialog between the participant and the examiner, during which open-ended questions were posed regarding topics such as local traffic, places of interest, and personal preferences. This format was designed to capture both structured and natural speech patterns.

Each audio file is labeled with metadata including the participant ID, Hoehn and Yahr (H&Y) stage, and Unified Parkinson’s Disease Rating Scale (UPDRS) scores for sections II-5 and III-18. For instance, the file name “ID02_pd_1_2_1.wav” refers to participant 02, diagnosed with PD at H&Y stage 1 (indicative of early-stage PD), with a UPDRS II-5 score of 2 and a UPDRS III-18 score of 1.

The dataset comprises recordings from a total of 37 participants, including 21 healthy controls and 16 individuals with PD. Due to the naturalistic conditions under which the speech samples were recorded and the inclusion of both read and spontaneous speech tasks, this dataset offers a highly authentic representation of real-world voice characteristics. Consequently, it is particularly well-suited for the objectives of the current research, which aims to investigate speech-based biomarkers for the detection of PD.

### 2.2. Methods

**Figure 1:**
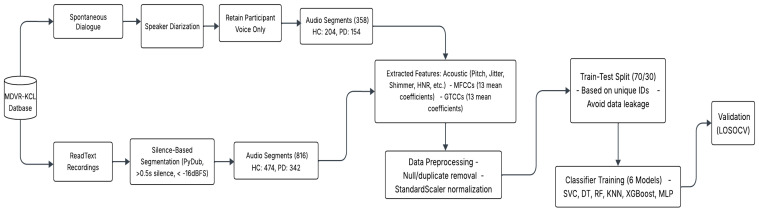
Structured graphs of audio signal processing, data processing, model building, and validation.

The proposed methodology is designed to detect and classify PD patients and distinguish HC from voice signals using ML. The implementation was carried out using Python 3. In Figure 1, we present a structured and formal description of each methodological step.

#### 2.2.1. Signal Processing

The MDVR-KCL dataset comprises two distinct types of audio recordings: read text and spontaneous dialog. The read text recordings consist solely of participants’ voices, while the spontaneous dialog involves two speakers, the participant and an examiner, engaged in a task-related conversation. Notably, the spontaneous dialog recordings contain overlapping speech, whereas the read text recordings feature single-speaker audio without overlapping. Given the structural differences between these two data types, separate preprocessing and feature extraction methods were required. Accordingly, two parallel processing pipelines were employed. To reduce variability across recordings, basic preprocessing steps were applied, including signal normalization and the use of mean feature aggregation over each audio segment. The extraction of MFCC and GTCC features inherently provides a degree of robustness to noise and recording variations, as these features capture perceptually relevant spectral characteristics. However, no advanced normalization or domain adaptation techniques were applied in this study. A brief description of each is provided below.

#### 2.2.2. Reading Text

This subset contains voice recordings from 37 participants (21 HC and 16 PD), with everyone contributing one recording per task. Due to the limited number of raw audio samples, data augmentation was performed by segmenting the recordings into smaller chunks. Segmentation was based on silence detection: an audio segment was split when at least 0.5 s of silence (defined as audio below −16 dBFS) was detected. This was implemented using *PyDub*, a Python library developed by James Robert [19]. As a result, 816 segmented audio files were generated, 474 from HC participants and 342 from PD participants.

#### 2.2.3. Spontaneous Dialog

In contrast to the *read text* data, the *spontaneous dialog* recordings feature overlapping speech between two speakers, rendering simple silence-based segmentation inadequate. Since the examiner’s speech is not relevant to the classification task, it was necessary to isolate the participant’s voice. To achieve this, a speaker diarization technique was performed using the pyannote.audio [20,21] library, enabling segmentation of the recordings into speaker-specific intervals. Only the segments corresponding to the participant’s voice were retained for analysis. This process yielded 358 usable audio segments: 204 segments from HC participants and 154 from PD participants.

It is important to emphasize that speaker diarization was crucial for ensuring the validity of the classification process in the *spontaneous dialog* task. Without this step, the inclusion of speech from the examiner, a healthy individual, could have introduced significant bias, thereby compromising the accuracy of the classification and potentially distorting the results.

### 2.3. Feature Extraction

In this study, we extracted Mel-Frequency Cepstral Coefficients (MFCCs), Gammatone Frequency Cepstral Coefficients (GTCCs), and a range of acoustic features from audio recordings. Feature extraction was performed using *Praat*, 6.4.65, a widely used software for phonetic and acoustic analysis, as introduced by Yannick Jadoul et al. [22]. The extracted features were saved in numerical format as CSV files for subsequent machine learning analysis.

#### 2.3.1. Acoustic Features

The acoustic features considered in this study reflect prosodic and phonatory alterations typically associated with PD. We consider those lower-level features following the study by Alalayah et al. [23]. These acoustic features include pitch, jitter, shimmer, and the harmonics-to-noise ratio (HNR). Pitch, or the fundamental frequency (F0), captures the average and variability of vocal fold vibration. HNR quantifies the proportion of harmonic sound to noise in the voice signal, with lower values indicating greater breathiness or roughness. Jitter and shimmer measure the cycle-to-cycle variation in frequency and amplitude, respectively, and are commonly used indicators of vocal instability in individuals with PD. The complete list of 11 extracted acoustic features is provided in the Supplementary File (see Table S1). To streamline discussions, we collectively refer to these parameters as acoustic features throughout the manuscript.

#### 2.3.2. MFCCs

MFCCs capture the short-term power spectrum of a speech signal and are modeled on the human auditory system’s response to sound. They are particularly effective in detecting articulation and phonation impairments caused by PD. The MFCC extraction process involves segmenting the audio signal into short time frames, applying the Fourier Transform, computing the logarithmic amplitude spectrum, and mapping it to the Mel scale, which reflects human pitch perception. A Discrete Cosine Transform (DCT) is then applied to obtain the final cepstral coefficients. In this study, we utilized the mean values of the first 13 MFCCs for each audio sample, including their delta (first-order derivative) components to capture temporal dynamics.

#### 2.3.3. GTCCs

GTCCs are derived using a gammatone filter bank, which simulates the filtering characteristics of human cochlea. This method offers a biologically plausible alternative to MFCCs, especially in capturing subtle pathological traits such as tremors or breathiness. The GTCC extraction process follows a similar pipeline to MFCCs but uses gammatone filters instead of Mel filters to enhance both temporal and spectral resolution. After applying the gammatone filter bank and computing the logarithmic magnitude of the filter outputs, the DCT is applied to generate the cepstral coefficients. As with MFCCs, we extracted the mean values of the first 13 GTCCs for our analysis.

### 2.4. Data Preprocessing

Following feature extraction from the audio signals, the numerical data were stored in CSV format. Data preprocessing is a critical step prior to model development and training in ML. The CSV files were imported into a Jupyter Notebook 7.5 using the *panda*’s library for further processing. Initial steps included exploratory data analysis involving graphical visualization and statistical summaries. Subsequently, the dataset was screened for missing and duplicate entries, which were removed to ensure data integrity. The classification target was defined by the “label” column, where a value of 1 indicated a PD case and 0 indicated an HC.

#### 2.4.1. Data Splitting

Splitting medical data requires caution to avoid data leakage. In this study, multiple audio-derived feature files were generated per participant following signal processing. Traditional data-splitting methods could inadvertently distribute data from the same individual across both training and testing subsets, potentially leading to model overfitting and biased performance estimates. To address this, we implemented a subject-wise split strategy. Unique participant identifiers were used to group all corresponding samples, ensuring that data from each subject were treated as a single unit during partitioning. These identifiers were then randomly shuffled to maintain class distribution before splitting.

Due to the limited dataset size, we opted for a two-way split: 70% of participants’ segments for training and 30% for the test set. Post-split inspection confirmed that no participant had data present in both sets. It is important to note that the dataset is imbalanced reflecting the typical distribution in real-world scenarios. Consequently, no data-balancing techniques (e.g., oversampling or under-sampling) were applied to preserve the ecological validity of the analysis.

#### 2.4.2. Standardization

Standardization is an essential preprocessing technique in ML, particularly for algorithms that are sensitive to feature scales. Many learning models, such as those using radial basis function (RBF) kernels in Support Vector Machines or regularization terms in linear models, assume that input features are approximately normally distributed with zero mean and unit variance. When features differ substantially in scale, those with larger variances can dominate the learning process, leading to suboptimal model performance. To mitigate this issue, we applied z-score normalization using the *StandardScaler* method from the *scikit-learn* library. This technique transforms each feature to have a mean of 0 and a standard deviation of 1, ensuring a standardized input space for all models.

### 2.5. Classification Models

We implemented six supervised learning algorithms for the binary classification of PD and HC subjects. To optimize model performance, we employed GridSearchCV, a systematic approach to hyperparameter tuning provided by the *scikit-learn* library. GridSearchCV performs an exhaustive search over a specified set of hyperparameter values by evaluating all possible combinations. For each combination, the model is trained and validated using *k*-fold cross-validation (*k* = 10), ensuring that the selected parameters generalize well across unseen data. The performance metric is computed for each fold, and the average score is used to rank the hyperparameter configurations. The set of parameters yielding the best average performance is then selected for final model evaluation. This approach reduces the risk of overfitting to the training dataset and ensures a fair comparison across different models and configurations. No test subject data was used during hyperparameter tuning. The Supplementary Material contains a list of the ML model name and parameters (see Table S2).

#### Validation Strategy

After selecting the optimal hyperparameters, the model performance was assessed using Leave-One-Subject-Out Cross-Validation (LOSOCV). This technique is particularly well-suited for small datasets. In this strategy, all samples from one subject are held out as the test set while the model is trained on the remaining subjects. This process is repeated for each subject, ensuring strict subject independence and preventing data leakage. LOSOCV is a widely adopted validation method in prior PD-related research, where dataset sizes are often limited. Its application in this study ensures rigorous and unbiased model evaluation.

## 3. Results

The performances of the ML models for each task are detailed here. Comprehensive metrics, including classification scores and class-centered values for both PD and HC cohorts, are provided in the Supplementary Materials for every experiment conducted in this study. Results pertaining to the reading task experiments are documented in Supplementary Material (see Tables S3–S8). Data from experiments involving spontaneous dialog are contained within Supplementary Materials (see Tables S10–S15).

To facilitate a rigorous performance comparison between different models and feature sets, statistical significance was evaluated using paired *t*-tests across the LOSOCV folds. This methodology was selected to account for fold-wise variability, ensuring a robust assessment of model efficacy. The analysis indicates whether the observed disparities between feature sets reached statistical significance based on calculated *p*-values. Detailed results of the paired *t*-test analyses are available in Supplementary Materials Tables S9 and S16.

### 3.1. Reading Task

In this study, we first evaluated the performance of various machine learning models for distinguishing PD from healthy controls (HCs) using recordings from a reading task. After applying the data preprocessing pipeline, we retained 566 training samples and 242 test samples. The training set consisted of 231 PD and 335 HC samples, while the test set included 108 PD and 134 HC samples. To find out the most relevant features from audio signals, we tested the ML model with independent and a combination of acoustic, MFCC, and GTCC features. First, we trained and tested ML models with those features separately. Then, we combined those features together, like, for example: acoustics with MFCCs and GTCCs; a combination of MFCC and GTCC coefficients; and lastly, a combination of acoustic MFCCs, and GTCCs. In the Supplementary Materials (see Tables S3–S8), we reported ML outcomes based on each feature set. We will highlight the best result for each feature set in main text. We will also highlight the best-performing model for each experiment.

#### 3.1.1. Individual Feature Set

In these experiments, we utilized each set of features to train and test ML models and their performance in classifying PD. In the Supplementary Materials, we add (see Supplementary Table S3) the ML model performance score in each feature set. In Supplementary Table S4, we add class-wise recall, precision, and F1-score.

***Acoustic Features****:* Using acoustic features alone, the RF model demonstrated the highest test accuracy at 81.82%, with a sensitivity of 71.43% and specificity of 80.08%. The F1-score was 74.71% with an AUC of 0.86 and an MCC of 0.62. The class-specific PD and HC are precision 0.78 and 0.84, recall was 0.71 and 0.88, and F1-score 0.75 and 0.86, respectively.

***MFCCs****:* Using MFCCs, the SVM model stood out, yielding a remarkable 91.43% accuracy, with 87.37% sensitivity, 94.00% specificity, 88.77% F1-score, AUC of 0.97, and MCC of 0.82. As per the PD and HC classes, precision was 0.90 and 0.88, recall was 0.80 and 0.95, and F1-score of 0.85 and 0.91, respectively. The KNN model also performed strongly, matching 90.20% accuracy while retaining high specificity (92.67%) and sensitivity (86.32%).

***GTCCs***: Using GTCCs, impressive performance was sustained across multiple models, with the MLP model delivering the highest accuracy (92.65%) alongside a sensitivity of 90.00% and specificity of 94.48%. This was further supported by an AUC of 0.98 and a strong MCC (0.85). For separate PD and HC classes, precision was 0.94 and 0.95, recall was 0.92 and 0.96, and F1-score was 0.93 and 0.95.

**Figure 2:**
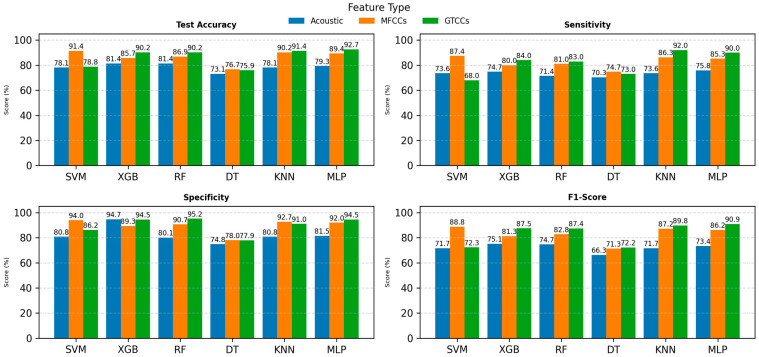
ML models’ performance comparison on individual feature sets. Abbreviations: SVM: Support Vector Machine; RF: Random Forest; XGB: Extreme Gradient Boosting; DT: Decision tree; KNN: K-Nearest Neighbors; MLP: Multilayer Perceptron; MFCCs: Mel-Frequency Cepstral Coefficients; and GTCCs: Gammatone Frequency Cepstral Coefficients.

Figure 2 presents a comparative performance analysis of SVM, XGB, RF, DT, KNN, and MLP using three feature sets: Acoustic features (blue), MFCCs (orange), and GTCCs (green). The evaluation metrics include test accuracy, sensitivity, specificity, and F1-score, with each depicted in separate subplots.

In the test accuracy plot (see Figure 2, top-left), the XGB and MLP models achieved the highest accuracy across feature types, with MFCCs generally outperforming acoustic and GTCC features. Sensitivity (see Figure 2, top-right) results show that MFCC-based models yielded superior performance, particularly for SVM (87.4%) and KNN (86.3.0%), indicating their stronger ability to correctly identify positive cases.

For specificity (see Figure 2, bottom-left), which measures the models’ ability to correctly classify negative cases, RF and MLP exhibited the highest values up to 95.2% and 94.5%, respectively, across most feature types. Meanwhile, F1-score (see Figure 2, bottom-right) results follow a similar trend, where MLP and XGB models demonstrate the best balance between precision and recall, particularly with MFCC and GTCC features.

Overall, the results highlight that MFCC and GTCC features consistently outperform general acoustic features across all performance metrics. Among classifiers, MLP achieved the most stable and superior results, suggesting its strong suitability for voice-based PD detection when combined with cepstral features.

#### 3.1.2. Combine Feature Sets

In these experiments, we combine two sets of features and evaluate ML models’ performance. Supplementary files show ML models’ results with feature combinations (see Table S5), and class-wise scores in Supplementary Table S6.

***Acoustic and MFCCs:*** Combining acoustic signals with MFCCs, the MLP model performed strongly with 91.32% accuracy, 89.00% sensitivity, 92.95% specificity, an F1-score of 89.75%, an AUC of 0.95, and an MCC of 0.82. When considering PD and HC classes separately, precision was 0.93 and 0.94, sensitivity was 0.92 and 0.95, and F1-score was 0.92 and 0.95, respectively. The XGBoost algorithm performed exceptionally well with 90.50% accuracy, 84.00% sensitivity (PD 0.91, HC 0.90), 95.07% specificity, an F1-score of 87.96% (PD 0.88, HC 0.92), and an AUC of 0.95.

**Acoustic and GTCCs:** When adding GTCCs to the acoustic signals, the SVM model performed exceptionally, yielding 95.45% accuracy, 94.62% sensitivity, 95.97% specificity, an F1-score of 94.12%, and an AUC of 0.98 with an MCC of 0.90. In separate PD and HC, class precision was 0.94 and 0.94, sensitivity was 0.89 and 0.97, and F1-score was 0.92 and 0.95, respectively. The MLP model closely followed, delivering 93.80% accuracy, 90.32% sensitivity, 95.97% specificity, an F1-score of 91.80%, and an AUC of 0.97 with an MCC of 0.87. Also, for PD and HC classes, precision: 0.93 and 0.93, sensitivity: 0.89 and 0.96, and F1-score: 0.91, 0.95.

**MFCCs and GTCCs**: When combining MFCCs with GTCCs, the KNN model performed well with 93.88% accuracy, 93.64% sensitivity (PD 0.91, HC 0.94), 94.07% specificity, an F1-score of 93.21% (PD 0.92, HC 0.93), and an AUC of 0.97 with an MCC of 0.88. The SVM model also performed strongly with 93.47% accuracy, 92.73% sensitivity, 93.07% specificity, an F1-score of 92.73%, and an AUC of 0.99 with an MCC of 0.87. For specific PD and HC classes, precision was 0.93 and 0.92, sensitivity was 0.89 and 0.97, and F1-score was 0.92 and 0.94, respectively.

**Figure 3:**
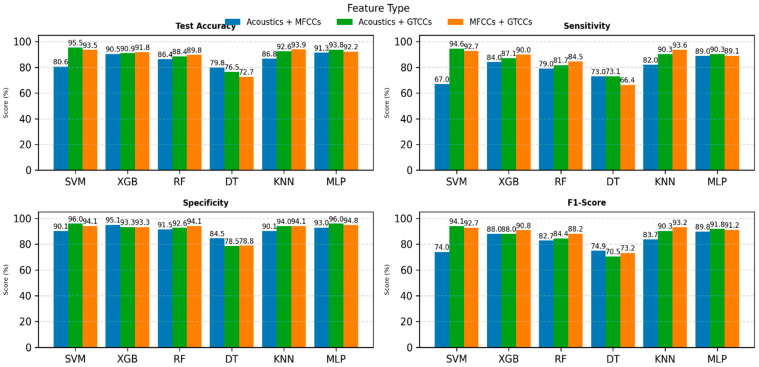
ML models’ performance on two combined feature sets. Abbreviations: SVM: Support Vector Machine; RF: Random Forest; XGB: Extreme Gradient Boosting; DT: Decision tree; KNN: K-Nearest Neighbors; MLP: Multilayer Perceptron; MFCCs: Mel-Frequency Cepstral Coefficients; and GTCCs: Gammatone Frequency Cepstral Coefficients.

Figure 3 illustrates comparative performance using combined feature sets derived from different acoustic and cepstral representations: Acoustics–MFCCs (blue), Acoustics–GTCCs (green), and MFCCs-GTCCs (orange). The models are evaluated based on four performance metrics: test accuracy, sensitivity, specificity, and F1-score, displayed across four subplots.

The top-left subplot (see Figure 3) shows test accuracy; the combination of GTCCs with the acoustic consistently yields the highest performance across most classifiers, particularly for SVM (≈95.5%) and MLP (≈93.8%). Sensitivity results (see Figure 3, top-right) show that the SVM and MLP models achieve superior recall rates, with the same feature combinations producing the best outcomes (94.6% for SVM and 90.3% for MLP and KNN), indicating strong capability in detecting positive samples.

For specificity (bottom-left of Figure 3), which reflects the ability to correctly identify HC, nearly all models maintain high performance (above 90%), with both MLP and SVM (96.0%), and again, the acoustic and GTCCs feature combination performs the best, demonstrating excellent discrimination between classes. Similarly, F1-score (see Figure 3, bottom-right) patterns align closely with accuracy and sensitivity trends, with the MLP and SVM models attaining the most balanced results between precision and recall, particularly when using combined cepstral features.

Overall, the Figure highlights that integrating cepstral and acoustic features enhances model robustness and generalization. Among the classifiers, the SVM and MLP models consistently deliver the most reliable and high-performing results across all metrics, emphasizing the effectiveness of hybrid feature fusion of acoustics and GTCCs for voice-based PD classification.

#### 3.1.3. Combination of All-Feature Sets

In the last experiment setup, we combine all features to train and evaluate the ML model performance. The ML model metric values for combined feature sets derived from different acoustic and cepstral representations are added in the Supplementary Files (see Tables S7 and S8). In the fusion of all-feature set, including acoustic, MFCCs and GTCCs, the MLP model achieved 93.80% accuracy with 90.91% sensitivity and 95.45% specificity. The F1-score was 91.43%, AUC was 0.98, and MCC was 0.87. The class-specific PD and HC had precision that was 0.95 and 0.96, sensitivity was 0.0.93 and 0.97, and an F1-score of 0.94 and 0.97 respectively. The KNN model performed identically, with 92.15% accuracy, 90.91% sensitivity (0.95 PD, 0.96 HC), 92.86% specificity, an F1-score of 89.39% (0.94 PD, 0.96 HC) and an AUC of 0.98 with an MCC of 0.83.

DT model exhibits the lowest performance, with test accuracy (78.5%) and sensitivity (69.3%), with PD of 0.69, and HC of 0.81, notably lagging the other model, suggesting overfitting or limited generalization capability. The RF and XGB models provide average performance, with RF achieving high specificity (94.8%) but slightly lower sensitivity (79.5%), indicating a bias toward correctly classifying HC.

**Figure 4:**
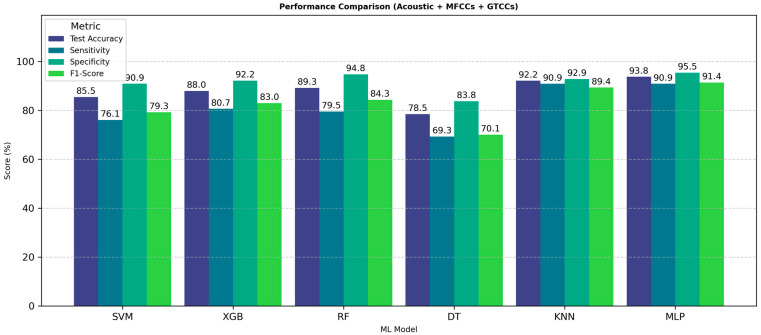
ML models’ performance with all features. Abbreviations: SVM: Support Vector Machine; RF: Random Forest; XGB: Extreme Gradient Boosting; DT: Decision tree; KNN: K-Nearest Neighbors; MLP: Multilayer Perceptron; MFCCs: Mel-Frequency Cepstral Coefficients; and GTCCs: Gammatone Frequency Cepstral Coefficients.

Figure 4 presents a performance analysis using the combined feature set of acoustic, MFCC, and GTCC features. In Figure 4, each bar shows the values of test accuracy, sensitivity, specificity, and F1-score of each model.

The results clearly demonstrate that the fusion of acoustic, MFCC, and GTCC features significantly enhances classification accuracy and reliability. Among all models, MLP achieves the most balanced and superior results, confirming its strong suitability for speech-based PD detection when using fusion of acoustic, and cepstral feature representations.

#### 3.1.4. ROC-AUC Curve (Read Text)

**Figure 5:**
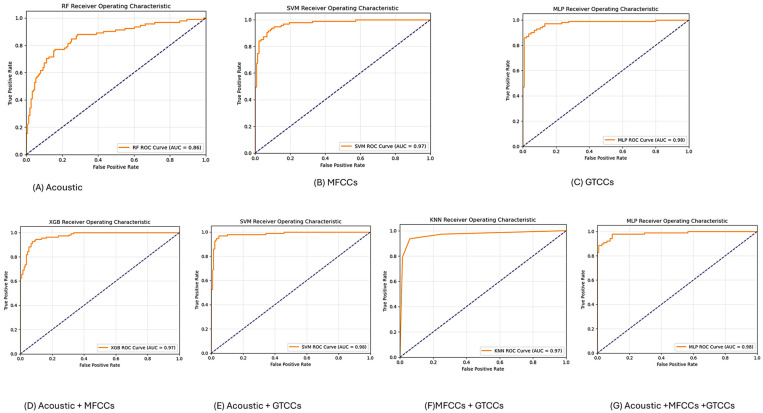
The ROC curve and AUC value from the Read-Text task experiments. Abbreviations: SVM: Support Vector Machine; RF: Random Forest; XGB: Extreme Gradient Boosting; DT: Decision tree; KNN: K-Nearest Neighbors; MLP: Multilayer Perceptron; MFCCs: Mel-Frequency Cepstral Coefficients; GTCCs: Gammatone Frequency Cepstral Coefficients; and AUC: Area under the curve.

In Figure 5, we add the ROC curve and the best AUC value of each experiment from our work. Here, panel (Figure 5A) represents the AUC of the RF model from acoustic features. Subfigure (Figure 5B) shows an AUC value of 0.97 from MFCC features by SVM. Subfigure (Figure 5C) shows an MLP model AUC value of 0.97 from GTCC values. In subfigure (Figure 5D), XGB was higher with an AUC value of 0.97 from the combined acoustic and MFCC features experiment. Subfigure (Figure 5E) shows the results of the experimental combination of acoustic and GTCCS, and it has the best AUC of 0.98, also by SVM. Subfigure (Figure 5F) shows an AUC of 0.97 by KNN from the MFCC and GTCC experiments. And subfigure (Figure 5G) shows an AUC value of 0.98 for the MLP model from the experiment combining of all the features.

### 3.2. Spontaneously Conversational Speech (Dialog)

In the spontaneous dialog condition, after applying the data preprocessing pipeline, we retained 214 training samples and 92 test samples. The training set consisted of 95 PD and 119 HC samples, while the test set included 36 PD and 56 HC samples. We followed the same procedure we mentioned in the spontaneous speech analysis. In the Supplementary Files (see Tables S10–S15), we reported ML outcomes based on each feature set. We will highlight the best results each feature set in the main text.

#### 3.2.1. Individuals Feature Set (Dialog)

In these experiments, we utilized each set of features to train and test ML models and their performance to classify PD. In the Supplementary Materials, we provided ML model performance in each feature set (see Table S10). The individual PD and HC class precision, F1-score, and sensitivity score of each model are added in Supplementary (see Table S11).

***Acoustic Features:*** Using acoustic signals alone, the RF model performed second best with a test accuracy of 70.65%, 47.36% sensitivity (PD 0.47, HC 0.89), 87.03% specificity, and an F1-score of 57.14% (PD 0.58, HC 0.79). The KNN model performed nearly identically with a 70.65% accuracy, 42.10% sensitivity (PD 0.47, HC 0.89), 90.74% specificity, and an F1-score of 54.23% (PD 0.58, HC 0.79). The XGBoost model performed slightly better with 75.00% accuracy, 44.74% sensitivity, 96.30% specificity, an F1-score of 59.65%, an AUC of 0.73, and an MCC of 0.50. For separate PD and HC classes, precision: 0.71 and 0.72, sensitivity: 0.53 and 0.85, and F1-score: 0.61 and 0.78, respectively.

**MFCCs:** Using MFCCs, the KNN model performed exceptionally well, with 80.77% accuracy, 70.00% sensitivity, 87.50% specificity, an F1-score of 73.68%, an AUC of 0.89, and an MCC of 0.59. And with individual PD and HC classes, precision scores were 0.78 and 0.82, respectively. Also, sensitivity values were 0.70 and 0.88, and F1-scores were 0.74 and 0.85, in the same order. The XGB model also performed strongly with 79.81% accuracy, 72.50% sensitivity (0.74 PD, 0.82 HC), 84.38% specificity, an F1-score of 73.42% (PD 0.72 and HC 0.83), an AUC of 0.83, and an MCC of 0.57.

**GTCCs:** Using GTCCs, the MLP model performed exceptionally with 81.98% accuracy, 82.98% sensitivity, 81.25% specificity, an F1-score of 79.59%, an AUC of 0.89, and an MCC of 0.64. For the class of independent PD, HC precision was 0.80 and 0.87, sensitivity was 0.83 and 0.84, and it had an F1-score of 0.81 and 0.86, respectively. The XGBoost model also performed well with 81.08% accuracy, 74.47% sensitivity, 85.94% specificity, an F1-score of 76.92%, an AUC of 0.88, and an MCC of 0.61. For each PD and HC class, precision was 0.73 and 0.83, sensitivity was 0.77 and 0.81, and F1-score was 0.76 and 0.84.

**Figure 6:**
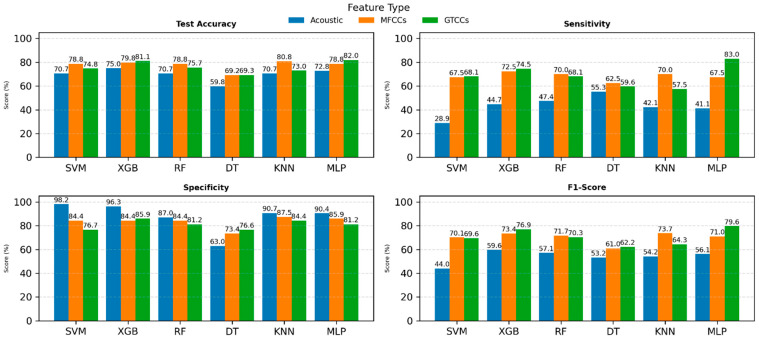
ML model performance with individual features set. Abbreviations: SVM: Support Vector Machine; RF: Random Forest; XGB: Extreme Gradient Boosting; DT: Decision tree; KNN: K-Nearest Neighbors; MLP: Multilayer Perceptron; MFCCs: Mel-Frequency Cepstral Coefficients; and GTCCs: Gammatone Frequency Cepstral Coefficients.

Figure 6 presents a performance analysis of SVM, XGB, RF, DT, KNN, and MLP using three feature sets: Acoustic features (blue), MFCCs (orange), and GTCCs (green) using spontaneous conversational speech records.

In the test accuracy plot (Figure 6, top-left), MLP models achieved the highest accuracy across feature types, with GTCC generally outperforming acoustic and MFCC features. Sensitivity (Figure 6, top-right) results show that MLP yielded a superior performance of 83% from GTCC features, indicating their stronger ability to correctly identify positive cases.

For specificity (Figure 6, bottom-left), which measures the models’ ability to correctly classify negative cases, SVM exhibited the highest values of up to 98% across most feature types. In the bottom-right of Figure 6, F1-score results show that the MLP and XGB models demonstrated the best balance between precision and recall, particularly with MFCC and GTCC features.

Overall, the results highlight that GTCC features consistently showed balanced performance against general acoustic and MFCC features across all performance metrics. Among classifiers, MLP achieved the most stable results, suggesting its strong suitability for voice-based PD detection when combined with cepstral features.

#### 3.2.2. Combine Feature Sets (Dialog)

**Figure 7:**
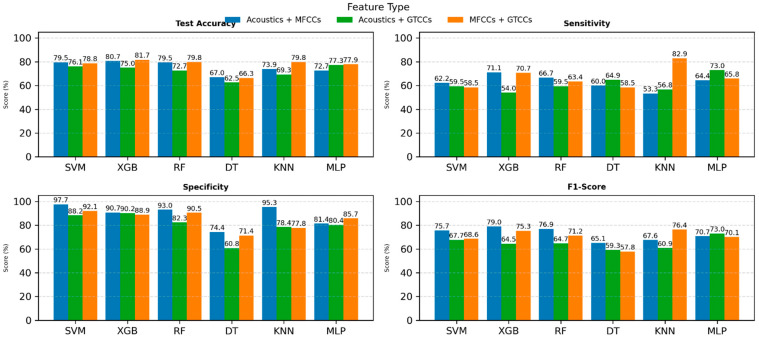
ML models’ performance comparison with a combination of two sets of features. Abbreviations: SVM: Support Vector Machine; RF: Random Forest; XGB: Extreme Gradient Boosting; DT: Decision tree; KNN: K-Nearest Neighbors; MLP: Multilayer Perceptron; MFCCs: Mel-Frequency Cepstral Coefficients; and GTCCs: Gammatone Frequency Cepstral Coefficients.

In these experiments, we combine two sets of features together to evaluate ML models’ performance. In the Supplementary Materials, we added the results of ML models with all features (see Tables S12 and S13). Figure 7 illustrates the comparative performance using combined feature sets derived from different acoustic and cepstral representations: Acoustics with MFCCs (blue), acoustics with GTCCs (green), and MFCCs with GTCCs (orange). The models are evaluated based on the following performance metrics: Test accuracy, sensitivity, specificity, and F1-score, displayed across four subplots.

***Acoustic and MFCCs:*** When combining acoustic signals with MFCCs, the XGBoost model performed strongly with 80.68% accuracy, 71.11% sensitivity, 90.70% specificity, an F1-score of 79.01%, an AUC of 0.90, and an MCC of 0.63. And for each PD and HC class, precision values were 0.85 and 0.76, sensitivity was 0.73 and 0.86, and they had F1-scores of 0.79 and 0.80. The RF model performed comparably with 79.54% accuracy, 66.67% sensitivity (PD 0.67, HC 0.95), 93.02% specificity, an F1-score of 76.92% (0.83 PD, 0.78 HC), an AUC of 0.92, and an MCC of 0.62.

***Acoustic and GTCCs:*** When adding GTCCs to the acoustic signals, the MLP model performed well with 77.27% accuracy, 72.97.46% sensitivity, 80.39% specificity, an F1-score of 72.97%, an AUC of 0.81, and an MCC of 0.53. When considering individual PD and HC, the precision score achieved 0.77 and 0.81, respectively; sensitivity was 0.73 for PD and 0.84 for HC, and the F1-score was 0.75 and 0.83, in that order. The SVM model also performed strongly with 76.14% accuracy, 59.46% sensitivity (0.59 PD, 0.88 HC), 88.24% specificity, an F1-score of 74.69% (PD 0.68, HC 0.81), an AUC of 0.82, and an MCC of 0.51.

**MFCCs and GTCCs:** When combining MFCCs with GTCCs, the XGBoost model performed well with 81.73% accuracy, 70.73% sensitivity, 88.89% specificity, an F1-score of 75.32%, an AUC of 0.89, and an MCC of 0.61. For individual PD and HC classes, precision values were 0.76 and 0.82, sensitivity was 0.71 and 0.86, and F1-score was 0.73 and 0.84. The RF model followed closely with 79.80% accuracy, 63.41% sensitivity, 90.47% specificity, an F1-score of 71.23%, an AUC of 0.88, and an MCC of 0.57. Also, precision was 0.74 and 0.80, sensitivity was 0.68 and 0.84, and F1-score was 0.71 and 0.82 for separate PD and HC.

The results highlight that integrating cepstral and acoustic features enhances model robustness and generalization. Among the classifiers, the XGB, KNN, and MLP models consistently deliver the most reliable and high-performing results across all metrics, emphasizing the effectiveness of hybrid feature fusion of acoustic and MFCCs for PD classification.

#### 3.2.3. All Feature Sets (Dialog)

In these experiments, we combine all features together to evaluate ML models’ performance. ML model results with feature combinations are included in the Supplementary Material (see Tables S14 and S15).

**Figure 8:**
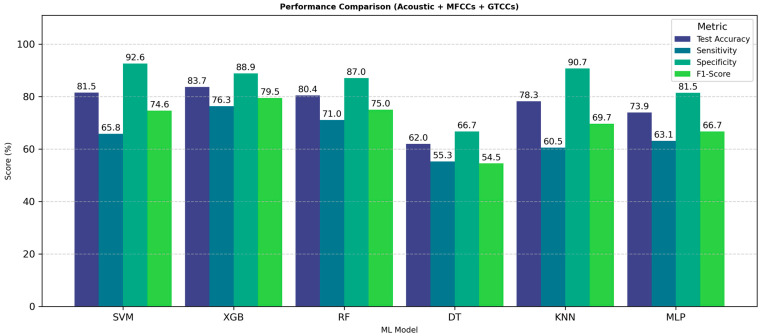
ML model performance comparison for PD identification from speech with combined feature sets. Abbreviations: SVM: Support Vector Machine; RF: Random Forest; XGB: Extreme Gradient Boosting; DT: Decision tree; KNN: K-Nearest Neighbors; MLP: Multilayer Perceptron; MFCCs: Mel-Frequency Cepstral Coefficients; and GTCCs: Gammatone Frequency Cepstral Coefficients.

**Acoustic, MFCCs, and GTCCs**: Finally, when combining all the feature sets, the XGBoost algorithm performed strongly with 83.70% accuracy, 76.32% sensitivity, 88.89% specificity, an F1-score of 79.45%, an AUC of 0.88, and an MCC of 0.66. The individual PD and HC class precision scores are 0.78 and 0.84, respectively. Also, sensitivity values are 0.76 and 0.85, and F1-scores are 0.77 and 0.84, in the same order. The RF model also performed well with 80.44% accuracy, 71.05% sensitivity, 87.04% specificity, an F1-score of 75.00%, an AUC of 0.85, and an MCC of 0.59. For separate PD and HC, precision was 0.74 and 0.79, sensitivity was 0.68 and 0.83, and F1-score was 0.71 and 0.81. Figure 8 shows performance analysis using the combined feature set of acoustic, MFCC, and GTCC features.

The XGB model demonstrates the highest overall performance across all metrics, achieving a test accuracy of 83.7%, sensitivity of 76.3.9%, specificity of 88.9%, and an F1-score of 79.5%, indicating excellent generalization and balanced classification capability. The RF model also performs competitively, with all metrics consistently above 70%, showing strong stability across evaluation criteria. Based on all metrics, value RF is the second best among other models.

The results exhibit that the fusion of acoustic, MFCC, and GTCC features significantly improves classification accuracy and reliability. Among all models, boosting classifier XGB achieves the most balanced results, confirming its strong suitability for speech-based PD detection when using cepstral feature representations with acoustic features.

#### 3.2.4. ROC-AUC Curve (Dialog)

**Figure 9:**
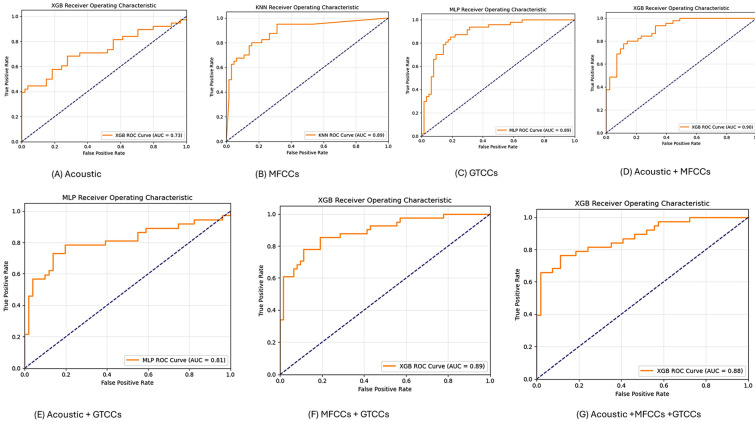
The ROC-AUC value from spontaneous dialog. Abbreviations: SVM: Support Vector Machine; RF: Random Forest; XGB: Extreme Gradient Boosting; DT: Decision tree; KNN: K-Nearest Neighbors; MLP: Multilayer Perceptron; MFCCs: Mel-Frequency Cepstral Coefficients; GTCCs: Gammatone Frequency Cepstral Coefficients; and AUC: Area under the curve.

Figure 9 shows the best AUC value of the best ROC curve of each experiment setup. Here, panel (Figure 9A) represents the AUC of the XGB model from acoustic features. Figure 9B shows the AUC value of 0.89 from the MFCC features by KNN. Figure 9C shows the MLP model AUC value of 0.89 from the GTCC values. In Figure 9D, XGB had a higher AUC value of 0.90 from the MFCC and acoustic combined feature experiment. Figure 9E shows the values for the experimental combination of acoustic and GTCC, and the best AUC of 0.81, which was also achieved by MLP. Figure 9F shows that AUC was 0.89 by XGB from the MFCC and GTCC experiments. And Figure 9G shows an AUC value of 0.88 for the XGB model from the combined experiment of all features.

## 4. Discussion

This study investigated the performance of various machine learning classifiers in distinguishing individuals with PD from healthy controls using both reading task recordings and spontaneous dialog, based on diverse feature sets, acoustic features, MFCCs, GTCCs, and their combination. The results demonstrate several key trends, highlighting the importance of feature representation, the type of speech, and the choice of classifier.

### 4.1. Effect of Feature Sets

Across both tasks among the single feature representations, the GTCC feature set model achieved superior performance compared to MFCC and acoustic features, particularly in terms of F1-score for PD subjects and ROC-AUC. The MFCC-based model also demonstrated strong performance, while acoustic features alone yielded comparatively lower results. While they capture prosodic and phonation-related cues (e.g., jitter, shimmer, and intensity), they lack the spectral richness of cepstral features, which may limit their discriminative power in isolation. MFCCs and GTCCs, being cepstral features, are effective in modeling the spectral characteristics of speech. GTCCs, which are more sensitive to glottal and pitch variations, appear particularly well-suited for PD detection when combined with MFCCs or acoustic features.

Feature fusion significantly improved classification performance. In particular, the combination of GTCC and acoustic features resulted in the highest overall performance, indicating that these features capture complementary information relevant to Parkinson’s disease detection. However, combining all feature sets did not consistently yield further improvements, suggesting potential redundancy and increased model complexity.

### 4.2. Task-Specific Observations

A critical observation is that classification performance was consistently higher for the reading task compared to spontaneous dialog. This can be attributed to several factors.

Controlled speech content in the reading task reduces lexical and prosodic variability, enabling more consistent acoustic pattern extraction across samples.

Spontaneous dialog introduces variability in speaking style, turn-taking, and emotion, making the detection of subtle PD-related speech impairments more challenging.

Additionally, speaker diarization errors and overlapping speech in spontaneous dialog may have degraded feature quality.

Nevertheless, despite the increased complexity of spontaneous dialog, classifiers trained on cepstral features and their combinations still achieved strong performance (up to 83.7% accuracy and 0.88 AUC with XGBoost). This confirms that robust PD speech markers can be extracted even from naturalistic conversations.

### 4.3. Statistical Significance Analysis

The performance of machine learning models was evaluated using different feature representations, including acoustic features, MFCCs, GTCCs, and their combinations. Across both the reading task and spontaneous dialog, hybrid feature sets generally achieved higher classification accuracy compared to individual feature groups. However, since several improvements were marginal, a paired *t*-test (α = 0.05) was conducted to evaluate whether performance differences between feature sets are statistically significant for both the reading task and spontaneous dialog, as shown in the Supplementary Materials (see Tables S9 and S16).

Reading Task: Acoustic vs. MFCCs/GTCCs: This was highly significant (*p* ≈ 0.0000) for most models, showing that acoustic features provide complementary information. MFCCs vs. GTCCs: Not significant (*p* > 0.05), indicating redundancy. MFCCs vs. Hybrid Features: Mostly not significant, with few exceptions (e.g., SVM, RF, DT, and MLP). Hybrid vs. All Features: Significant improvements mainly for RF, KNN, and SVM, suggesting ensemble models benefit most from feature fusion.

Spontaneous Dialog: Acoustic vs. MFCCs/GTCCs: Still significant across most models, confirming robustness of acoustic features. MFCCs vs. GTCCs: Mostly not significant, except KNN (*p* = 0.0014). MFCCs vs. Hybrid Features: Largely not significant, with minor improvements (MLP). Hybrid vs. All Features: Fewer significant results; several *p*-values = 1.0000 indicate negligible differences.

Acoustic features are consistently significant across both tasks, highlighting their importance in PD detection. MFCCs and GTCCs are redundant, contributing similar spectral information. Feature fusion improves accuracy, but gains are not always statistically significant. Reading speech shows stronger significance than spontaneous speech, due to lower variability. Model dependency exists: Random Forest benefits most from feature fusion, while XGBoost and MLP show minimal sensitivity.

Overall, combining MFCCs, GTCCs, and acoustic features yields the best performance, but statistical tests show that improvements are not always significant, especially in spontaneous speech. A balanced approach between feature complexity and model selection is therefore recommended.

### 4.4. Bias Analysis

Per-class evaluation revealed that some models exhibited lower recall for PD subjects compared to healthy controls, indicating potential bias toward the majority class. Notably, GTCC-based and fused feature models demonstrated improved sensitivity for PD detection, which is critical for clinical applications where minimizing false negatives is essential.

### 4.5. Performance Comparison with Previously Published Studies

In this subsection, we compare the performance of our study with previous research using the MDVR-KCL dataset. Through a comprehensive literature review, we identified six studies that have utilized this dataset. However, two of these studies by Rohit et al. [17] and Huang et al. [18] are not open access, and therefore, we could not access the methodological details or reported results. Consequently, our comparative analysis is limited to the remaining four studies, for which sufficient information is publicly available. Table 1 presents a summary of the methods, features, and best-reported performances of these studies.

Di Cesare et al. [15] utilized both reading text and conversational speech tasks in their study. They applied speaker diarization techniques to remove overlaps between the interviewer and participants in the conversational speech recordings. Following this, they implemented a sampling strategy on both types of audio signals, although the sampling methodology was not clearly described. Their feature set included MFCCs and GTCCs. The best performance was achieved using a K-Nearest Neighbors (KNN) classifier on the read-text task, yielding an accuracy of 92.3%, sensitivity of 93%, specificity of 91%, and an F1-score of 92. For the conversational speech, the highest performance was obtained using a Support Vector Machine (SVM), with similar metrics except for an F1-score of 90. Acoustic features were not included in their analysis, and they did not report AUC or MCC values.

Klempíř et al. [16] focused solely on read-text speech signals. They used the wav2vec 1.0 method and extracted only MFCC features. Their study reported an AUC of 0.72 using a Random Forest classifier and 0.78 with XGBoost. Other performance metrics were not disclosed in their publication.

Yousif et al. [13] also analyzed read text (spontaneous speech) recordings. Their approach involved both numerical and graphical processing of voice signals. They analyzed spectrograms, Mel-spectrograms, STFT, and MFCC features (using both Slaney and HTK toolkits). Supervised machine learning models applied to these features achieved near-perfect results using KNN, with reported accuracy, sensitivity, specificity, F1-score, and AUC all approaching 100%. Graphical features were evaluated using pre-trained convolutional neural networks (CNNs), where the best performance was obtained using the VGG16 model, with an accuracy, sensitivity, specificity, and F1-score of 96.93%, and an AUC of 99.55%.

Reddy and Akku [15] analyzed both spontaneous speech and dialog using a sparse learning approach. They did not specify any sampling technique or strategy for managing overlapping speakers. Their analysis was limited to MFCC features. Their proposed model, NSRC, demonstrated strong results on the read-text task, achieving an accuracy of 82.46%, sensitivity of 89.24%, precision of 82.73%, F1-score of 86.14, and MCC of 0.50. For the spontaneous dialog task, the model slightly outperformed traditional methods with an accuracy of 83.08%, sensitivity of 82.46%, precision of 79.66%, F1-score of 81.03, and MCC of 0.57.

**Figure 10:**
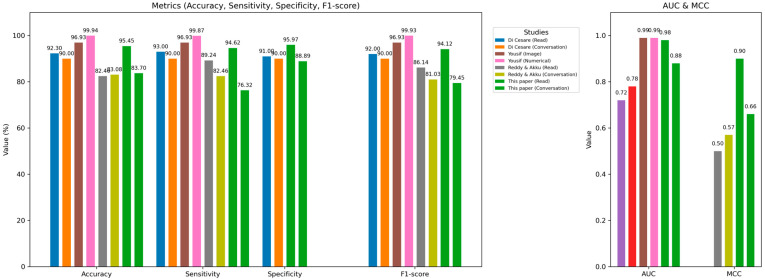
Performance comparison of the MDVR-KCL dataset relevant to this study. The left subfigure shows studies of metrics, accuracy, sensitivity, specificity, and F1-score. The subplot on the right illustrates AUC and MCC values. The values of this study are in green-colored bars. Abbreviations: MCC: Matthew’s Correlation Coefficient; and AUC: Area under the curve.

Figure 10 shows a bar chart comparison of each study’s results. The subplot on the left shows metric values, and the subplot on the right shows the AUC and MCC values of each study.

In comparison, our study is distinct in both methodological design and feature selection. We analyzed all available recordings from both read text and spontaneous dialog tasks, incorporating acoustic features alongside MFCCs and GTCCs for machine learning model development. We trained six different ML models. Our best performance on the read-text task was achieved using an SVM with an accuracy of 93.46%, sensitivity of 90.90%, precision of 95.20%, F1-score of 91.84, AUC of 0.97, and MCC of 0.86. For the spontaneous dialog task, the best result was obtained with the XGBoost model, achieving an accuracy of 83.70%, sensitivity of 76.32%, precision of 88.89%, F1-score of 79.45, AUC of 0.88, and MCC of 0.66.

In medical diagnosis tasks, especially those involving imbalanced datasets such as PD detection, precision and sensitivity are critical for evaluating true positive predictions. Relying solely on accuracy can be misleading. The F1-score is particularly informative in such contexts, as it balances precision and recall. AUC is a valuable metric for comparing models on the same dataset, while MCC provides an overall measure of model performance that considers all confusion matrix categories. Our results indicate a balanced and effective approach for both speech tasks using a comprehensive feature set and robust model evaluation.

### 4.6. Study Limitations and Future Work

The primary limitation of this study is the relatively small sample size. A larger cohort of participants during data acquisition could have significantly influenced methodological choices and led to more generalizable and robust results. The slightly lower performance observed in the spontaneous conversation task can be attributed to the limited number of samples; it is likely that increasing the number of subjects would improve model performance.

However, it is important to acknowledge that applying the same methodological framework to a larger and more heterogeneous dataset, without adequate adjustments and a rigorous training phase, may result in diminished performance. This underscores the need for carefully tailored training strategies when scaling up, and it raises important questions about the nature and complexity of the training required to maintain high model performance across broader populations.

Although this study combines multiple feature types, the overall feature dimensionality remains relatively low. Specifically, we utilized mean values of the first 13 MFCCs, 13 GTCCs, and 11 acoustic features, resulting in a total of 37 features per sample. Given this manageable feature size, explicit feature selection or dimensionality reduction techniques were not applied. However, combining heterogeneous feature sets may still introduce a risk of overfitting, particularly with limited data. To avoid this, we employed cross-validation (LOSOCV) and regularized ML models to ensure robust generalization. We also acknowledge that external validation is essential for assessing generalizability. Due to the limited availability of compatible datasets, independent cohort validation was not performed in this study. Future work will focus on evaluating the proposed framework on larger and multi-center datasets and will explore feature selection and dimensionality reduction methods, such as Recursive Feature Elimination (RFE) and Principal Component Analysis (PCA), to further improve model performance and interpretability.

Another limitation of this study is the lack of standardization in the selection of speech passages. Variability in linguistic complexity, phonetic richness, emotional tone, passage length, and familiarity can significantly affect speech production and acoustic features. All factors that influence speech production should be systematically controlled in future experimental designs. Future work will address this by incorporating controlled and standardized speech material, along with phonetic-level analysis, such as vowel space area and consonant articulation patterns, to better account for variability across participants.

These challenges reflect real-world conditions, where background noise, overlapping speech, and variable conversational dynamics are common. Such complexities may affect feature stability and model performance. Although MFCC and GTCC features are widely recognized for their robustness in speech analysis, variability in recording conditions, like device differences and environmental noise, can still influence their reliability, particularly in uncontrolled settings. Addressing these challenges will require more advanced speaker diarization techniques and the extraction of richer feature sets, including extended cepstral, prosodic, voice quality, and articulatory features. These enhancements may improve model robustness and accuracy in complex acoustic environments and support more reliable real-world deployment.

Deep learning approaches, such as CNNs, have shown strong performance in speech-based disease detection; they typically require large-scale datasets to achieve reliable generalization. In this study, the relatively limited dataset size motivated the use of classical machine learning models, which are better suited for small, structured feature sets and offer greater robustness and interpretability under such conditions. Nevertheless, deep learning remains a promising direction for future research. With the availability of larger and more diverse datasets, future work will explore lightweight architectures, such as 1D CNNs applied to MFCC or GTCC features, as well as hybrid approaches combining deep and handcrafted features to further enhance performance.

While the proposed models achieved strong classification performance, clinical interpretability remains limited, as the current analysis focuses primarily on predictive accuracy rather than the contribution of individual features. Features related to voice stability (e.g., jitter and shimmer) and cepstral representations (MFCCs and GTCCs) are likely to play an important role in classification, given their relevance to phonatory and articulatory impairments in PD. Future work will incorporate feature importance analyses, such as SHAP or model-based importance measures, to improve clinical insight.

This study is limited to binary classification (PD vs. HC), which restricts its applicability for monitoring disease progression. Future research will extend this framework to predict disease severity (e.g., MDS-UPDRS scores) and model longitudinal progression, subject to the availability of longitudinal clinical data. The current work relies solely on speech-based biomarkers. Integrating speech features with complementary modalities, such as neuroimaging biomarkers, may further improve diagnostic performance and pathophysiological interpretability. However, access to such medical imaging data is often limited due to privacy concerns and the lack of publicly available datasets. Future work will explore multimodal frameworks incorporating imaging and clinical data, contingent upon the availability of appropriate datasets or the ability to collect such data in compliance with ethical and regulatory requirements.

It should also be emphasized that this study provides only a preliminary, voice-based assessment of Parkinson’s disease and is not intended to substitute for clinical evaluations conducted by healthcare professionals, such as those based on the MDS-UPDRS. Nonetheless, this research represents a promising step toward automated, real-time health monitoring through voice analysis. A future implementation, potentially via a mobile application, could support both in-home and clinical use cases. However, such applications must rigorously address issues of data privacy, informed consent, and regulatory compliance in accordance with frameworks such as the GDPR and HIPAA.

In summary, this study highlights the potential of cepstral features and machine learning models in the detection of PD and lays important groundwork for future applications in the diagnosis and monitoring of neurodegenerative conditions through speech-based analysis.

## 5. Conclusions

This study explored the application of machine learning techniques to the automatic detection of PD using voice recordings from spontaneous speech and conversation. By extracting and combining multiple feature types, including acoustic parameters, Mel-Frequency Cepstral Coefficients (MFCCs), and Gammatone Cepstral Coefficients (GTCCs), we developed and evaluated classifiers capable of distinguishing PD patients from healthy controls with strong performance across multiple metrics.

Our results show that the combination of cepstral and acoustic features consistently improves classification accuracy, sensitivity, and robustness, compared to using any single feature type. The best performances were achieved using an SVM model on the reading task (accuracy: 95.45%, AUC: 0.98, and MCC: 0.90) and an XGBoost model on spontaneous dialog (accuracy: 83.70%, AUC: 0.88, and MCC: 0.66). These findings indicate that relevant vocal biomarkers of PD are present even in unstructured, conversational speech, though performance is somewhat reduced due to its inherent variability.

Nonetheless, the study is constrained by certain limitations, including a limited sample size, a lack of standardized speech content, and difficulties in managing overlapping speech during dialog. Addressing these challenges in future research through larger and more diverse datasets, advanced diarization techniques, and deep learning models capable of capturing temporal and contextual dependencies will be crucial for improving system performance and real-world applicability.

Future work will explore cross-linguistic generalization by evaluating the proposed models on speech data from native Italian speakers. Specifically, one dataset will be used for training and another for testing in an inter-database evaluation setup. This approach will help assess the models’ generalizability across different languages and recording conditions. However, the limited size of available datasets may still pose challenges for training high-capacity models, making transfer learning or data augmentation valuable strategies to explore.

Overall, this work demonstrates the feasibility of using voice as a non-invasive, accessible biomarker for PD detection and monitoring. It contributes to the growing body of evidence supporting speech-based digital biomarkers and lays the groundwork for the development of real-time, mobile, and telehealth-compatible screening tools for neurodegenerative diseases.

## Figures and Tables

**Table 1:** Comparison of the selected literature classification results obtained from the MDVR-KCL dataset that are relevant to the results obtained in this study.

Study	Audio Signal	Method	Features	ML/DL Technique	Best Outcome					
					Acc	Sen	Spe	F1	AUC	MCC
Di Cesare et al. [14]	Read Text	Sampling	MFCC, GTCC	KNN	92.3	0.93	0.91	0.92	N/A	N/A
Di Cesare et al. [14]	Conversation	Speaker Diarization	MFCC, GTCC	SVM	90	0.90	0.90	0.90		N/A
Klempíř et al. [16]	Read Text	Wav2Vec 1.0	MFCC	RF					0.72	N/A
Klempíř et al. [16]	Read Text	Wav2Vec 1.0	MFCC	XGB					0.78	N/A
Yousif et al. [13]	Read Text	Image	Spectrogram, Mel-spectrogram, STFT, and MFCC (Slaney and HTK)	Pre-trained CNN VGG16	96.93	96.93		96.93	99.55	N/A
Yousif et al. [13]	Read Text	Numerical		KNN	99.94	99.87		99.93	99.93	N/A
Reddy and Akku [15]	Read Text	Sparse	MFCC	NSRC	82.46	89.24		86.14		0.50
Reddy and Akku [15]	Conversation	Sparse	MFCC	NSRC	83.08	82.46		81.03		0.57
This Study	Read Text	Sampling	Acoustics, GTCCs	SVM	95.45	94.62	95.97	94.12	0.98	0.90
This Study	Conversation	Speaker diarization, sampling	Acoustic, MFCCs, GTCCs	XGB	83.70	76.32	88.89	79.45	0.88	0.66

Abbreviations: SVM: Support Vector Machine; RF: Random Forest; XGB: Extreme Gradient Boosting; KNN: K-Nearest Neighbors; CNN: Conventional neural network; MCC: Matthew’s Correlation Coefficient; AUC: Area under the curve; MFCCs: Mel-Frequency Cepstral Coefficients; GTCCs: Gammatone Frequency Cepstral Coefficients; ACC: Accuracy; Sen: Sensitivity; and Spe: Specificity.

## Data Availability

The original data presented in the study are openly available in the EU Open Research Repository Zenodo at DOI: https://doi.org/10.5281/zenodo.2867216. Accessed on 28 March 2026.

## Supplementary Materials

The following supporting information can be downloaded at: https://www.mdpi.com/article/10.3390/neurolint18050088/s1, Table S1: Extracted acoustic features list; Table S2: Models and Parameters; Table S3: Summarizes the performance metrics for each classifier across the different feature sets (Reading task); Table S4: The performance of ML Models on Individuals PD and HC class (Reading task); Table S5: Summarizes the performance metrics for each classifier across the different feature sets (Reading task); Table S6: Individuals PD and HC class values in feature Combinations (Reading task); Table S7: ML model performance with all features set (Reading task); Table S8: Individuals PD and HC class values in all features (Reading task); Table S9: Features comparisons paired with *t*-test score of ml model (Reading task); Table S10: The performance metrics for each ML model across the different feature sets Spontaneous dialog; Table S11: Individuals PD and HC class values in features independent (Spontaneous dialog); Table S12: ML models performance with combination of two sets of features (Spontaneous dialog); Table S13: Individuals PD and HC class values in features combined (Spontaneous dialog); Table S14: ML models performance for PD identification from speech with Combine Feature sets (Spontaneous dialog); Table S15: Individuals PD and HC class values in all features combined (Spontaneous dialog); Table S16: Features comparisons paired with *t*-test score of ml model (Spontaneous dialog).
