# Supplementary material for: Machine Learning Approaches to Early Detection of Parkinson’s Disease Using Speech Analysis Technique
Source: Neurol Int. 2026 May 10;18(5):88. doi: 10.3390/neurolint18050088 (PMC13209858; doi:10.3390/neurolint18050088)
Supplement: Supplementary file 1 [file neurolint-18-00088-s001.zip › neurolint-4261418-supplementary.pdf]

Supplementary File

Machine learning Approaches to early detection of Parkinson's disease using speech analysis technique.

Mohammad Amran Hossain*[1], Enea Traini[1], and Francesco Amenta[2]

1. Telemedicine and Telepharmacy Centre, School of Medicinal and Health Products Sciences, University of Camerino, 62032 Camerino, Italy.
2. Research Department, International Radio Medical Centre (C.I.R.M.), 00144 Rome, Italy

* Correspondence: mohammad.hossain@unicam.it

Abbreviations

SVM: Support Vector Machine.
RF: Random Forest.
XGB: Extreme Gradient Boosting.
DT: Decision tree.
KNN: K-Nearest Neighbors.
MLP: Multilayer Perceptron.
MCC: Matthew's Correlation Coefficient.
AUC: Area under the Curve.
MFCCs: Mel-Frequency Cepstral Coefficients.
GTCCs: Gammatone Frequency Cepstral Coefficients.

Table S 1: Extracted acoustic features list

**Feature Name**	**Description**	**Type**
Pitch: F0 (Hz) - Mean	Mean of the fundamental frequency	Numerical
Pitch: F0 (Hz) - SD	Standard deviation of the fundamental frequency	Numerical
HNR	Mean harmonics-to-noise ratio	Numerical
Jitter: local	Local variation in pitch	Numerical
Jitter: absolute	Absolute jitter	Numerical
Jitter: RAP	Relative average perturbation	Numerical
Jitter: PPQ5	Five-point period perturbation quotient	Numerical
Shimmer: local	Local variation in amplitude	Numerical
Shimmer: dB	Shimmer in decibels	Numerical
Shimmer: APQ3	Three-point amplitude perturbation quotient	Numerical
Shimmer: APQ5	Five-point amplitude perturbation quotient	Numerical

Table S 2: Models and Parameters

Model name	Parameters
Support Vector Machine (SVM)	C=10, gamma='auto', probability=True
Decision Tree (DT)	max_depth=10, max_features='sqrt', min_samples_split=5, random_state=42
Random Forest (RF)	n_estimators=200, random_state=42
K-Nearest Neighbors (KNN)	metric='manhattan', n_neighbors=7
XGBoost Classifier	colsample_bytree=1.0, eval_metric='mlogloss', learning_rate=0.1, n_estimators=100, random_state=42
Multilayer Perceptron (MLP)	alpha=0.001, hidden_layer_sizes=(50,), max_iter=300, random_state=42

Table S 3: Summarizes the performance metrics for each classifier across the different feature sets (Reading task)

Feature	ML Model	Train Accuracy	Test Accuracy	Sensitivity	Specificity	F1-Score	AUC	MCC
Acoustic	SVM	84.45	78.10	73.63	80.79	71.66	0.82	0.49
	XGB	91.52	81.40	74.73	94.65	75.14	0.86	0.54
	**RF**	**99.82**	**81.82**	**71.43**	**80.08**	**74.71**	**0.86**	**0.62**
	DT	100	73.14	70.33	74.83	66.32	0.77	0.45
	KNN	100	78.10	73.63	80.79	71.66	0.85	0.59
	MLP	95.76	79.34	75.82	81.46	73.40	0.84	0.43
MFCCs	**SVM**	**94.57**	**91.43**	**87.37**	**94.00**	**88.77**	**0.97**	**0.82**
	XGB	84.58	85.71	80.00	89.33	81.28	0.93	0.69
	RF	86.16	86.94	81.05	90.67	82.79	0.91	0.71
	DT	76.18	76.73	74.73	78.00	71.35	0.77	0.52
	KNN	91.59	90.20	86.32	92.67	87.23	0.96	0.79
	MLP	89.84	89.39	85.26	92.00	86.17	0.94	0.77
GTCCs	SVM	76.35	78.78	68.00	86.21	72.34	0.83	0.55
	XGB	87.04	90.20	84.00	94.48	87.50	0.95	0.79
	RF	87.22	90.20	83.00	95.17	87.36	0.95	0.79
	DT	75.48	75.91	73.00	77.93	72.22	0.75	0.50
	KNN	90.36	91.43	92.00	91.03	89.76	0.97	0.82
	**MLP**	**94.28**	**92.65**	**90.00**	**94.48**	**90.91**	**0.98**	**0.85**

Table S 4: The performance of ML Models on Individuals PD and HC class (Reading task)

Feature Name	ML Model	Precision		Sensitivity/Recall		F1- Score	
		PD	HC	PD	HC	PD	HC
Acoustic	SVM	0.71	0.83	0.71	0.82	0.71	0.82
	XGB	0.74	0.85	0.75	0.84	0.74	0.84
	RF	0.78	0.84	0.71	0.88	0.75	0.86

	DT	0.61	0.80	0.69	0.73	0.65	0.76
	KNN	0.69	0.85	0.76	0.79	0.72	0.83
	MLP	0.71	0.85	0.76	0.81	0.73	0.83
MFCC	SVM	0.90	0.88	0.80	0.95	0.85	0.91
	XGB	0.88	0.85	0.81	0.91	0.83	0.89
	RF	0.87	0.88	0.80	0.93	0.84	0.90
	DT	0.79	0.80	0.67	0.81	0.68	0.80
	KNN	0.87	0.91	0.86	0.92	0.87	0.92
	MLP	0.89	0.92	0.87	0.93	0.88	0.93
GTCC	SVM	0.96	0.94	0.91	0.97	0.93	0.96
	XGB	0.91	0.91	0.87	0.94	0.89	0.93
	RF	0.94	0.89	0.82	0.97	0.88	0.92
	DT	0.69	0.78	0.67	0.79	0.68	0.78
	KNN	0.88	0.94	0.92	0.91	0.90	0.93
	MLP	0.94	0.95	0.92	0.96	0.93	0.95

Table S 5: Summarizes the performance metrics for each classifier across the different feature sets (Reading task)

Feature	ML Model	Train Accuracy	Test Accuracy	Sensitivity	Specificity	F1-Score	AUC	MCC
Acoustics + MFCCs	SVM	82.69	80.58	67.00	90.14	74.03	0.88	0.59
	XGB	88.52	90.50	84.00	95.07	87.96	0.95	0.80
	RF	86.75	86.36	79.00	91.45	82.72	0.93	0.72
	DT	78.27	79.75	73.00	84.50	74.87	0.78	0.57
	KNN	90.99	86.78	82.00	90.14	83.67	0.95	0.72
	**MLP**	**90.64**	**91.32**	**89.00**	**92.95**	**89.75**	**0.95**	**0.82**
Acoustics + GTCCs	**SVM**	**92.40**	**95.45**	**94.62**	**95.97**	**94.12**	**0.98**	**0.90**
	XGB	86.57	90.91	87.10	93.29	88.04	0.96	0.80
	RF	87.10	88.43	81.72	92.61	84.44	0.94	0.75
	DT	76.15	76.45	73.12	78.52	70.46	0.76	0.51
	KNN	91.16	92.56	90.32	93.96	90.32	0.97	0.84
	MLP	91.69	93.80	90.32	95.97	91.80	0.97	0.87
MFCCs + GTCCs	SVM	99.82	93.47	92.73	94.07	92.73	0.99	0.87
	XGB	100	91.84	90.00	93.33	90.83	0.97	0.83
	RF	100	89.80	84.55	94.07	88.15	0.97	0.79
	DT	97.39	72.65	66.36	78.77	73.17	0.79	0.54
	**KNN**	**99.67**	**93.88**	**93.64**	**94.07**	**93.21**	**0.97**	**0.88**
	MLP	99.30	92.24	89.09	94.81	91.16	0.97	0.84

Table S 6: Individuals PD and HC class values in feature Combinations (Reading task)

Feature Name	ML Model	Precision		Sensitivity/Recall		F1- Score	
		PD	HC	PD	HC	PD	HC
	SVM	0.95	0.87	0.80	0.97	0.87	0.92
	XGB	0.91	0.90	0.86	0.94	0.88	0.92

Acoustics + MFCCs	RF	0.90	0.85	0.77	0.94	0.83	0.89
	DT	0.76	0.82	0.74	0.83	0.75	0.83
	KNN	0.85	0.91	0.88	0.89	0.87	0.90
	MLP	0.93	0.94	0.92	0.95	0.92	0.95
Acoustics + GTCCs	SVM	0.94	0.94	0.89	0.97	0.92	0.95
	XGB	0.89	0.95	0.91	0.93	0.90	0.94
	RF	0.85	0.89	0.82	0.91	0.84	0.90
	DT	0.61	0.81	0.73	0.71	0.67	0.76
	KNN	0.91	0.94	0.90	0.95	0.91	0.94
	MLP	0.93	0.93	0.89	0.96	0.91	0.95
MFCCs + GTCCs	SVM	0.96	0.92	0.89	0.97	0.92	0.94
	XGB	0.90	0.89	0.85	0.92	0.87	0.90
	RF	0.91	0.89	0.86	0.93	0.89	0.91
	DT	0.77	0.80	0.75	0.81	0.76	0.81
	KNN	0.91	0.94	0.93	0.93	0.92	0.93
	MLP	0.94	0.94	0.93	0.96	0.94	0.95

Table S 7: ML model performance with all features set (Reading task)

Feature	ML Model	Train Accuracy	Test Accuracy	Sensitivity	Specificity	F1-Score	AUC	MCC
Acoustic +MFCCs +GTCCs	SVM	89.05	85.54	76.14	90.91	79.29	0.90	0.68
	XGB	100	88.02	80.68	92.21	83.04	0.95	0.74
	RF	100	89.26	79.55	94.81	84.34	0.96	0.77
	DT	100	78.51	69.32	83.77	70.11	0.77	0.53
	KNN	94.88	92.15	90.91	92.86	89.39	0.98	0.83
	**MLP**	**100**	**93.80**	**90.91**	**95.45**	**91.43**	**0.98**	**0.87**

Table S 8: Individuals PD and HC class values in all features (Reading task)

Feature Name	ML Model	Precision		Sensitivity/Recall		F1- Score	
		PD	HC	PD	HC	PD	HC
Acoustic +MFCCs +GTCCs	SVM	0.93	0.94	0.90	0.96	0.91	0.95
	XGB	0.83	0.89	0.80	0.91	0.81	0.90
	RF	0.86	0.90	0.82	0.92	0.84	0.91
	DT	0.67	0.82	0.69	0.81	0.68	0.81
	KNN	0.95	0.96	0.92	0.97	0.94	0.96
	MLP	0.95	0.96	0.93	0.97	0.94	0.97

Table S 9: Features comparisons paired with t-test score of ml model (Reading task)

ML Model / P - value	Acoustic vs MFCCs	Acoustic vs GTCCs	MFCCs vs GTCCs	MFCCs vs MFCCs+ GTCCs	MFCCs vs MFCCs +Acoustic	MFCCs vs GTCCs+ Acoustic	MFCCs + Acoustic vs ALL	GTCCs + Acoustic vs ALL	GTCCs + MFCCs vs ALL
SVM	0.0000	0.0000	0.3844	0.0389	0.4655	0.2209	0.0412	0.4389	0.2255
XGB	0.0000	0.0000	0.5154	0.2173	0.3307	0.4661	0.5903	0.1710	0.8528
RF	0.0000	0.0000	0.5997	0.1442	0.1236	0.0222	0.0195	0.0000	0.3431
DT	0.7152	0.1902	0.0969	0.0017	0.289	0.1728	0.4907	0.115	0.7740
KNN	0.0000	0.0000	0.3844	0.0881	0.4331	0.1167	0.0044	0.3461	0.8187
MLP	0.000	0.000	0.2501	0.0412	0.0864	0.8350	0.3941	0.1799	0.3176

Table S 10: The performance metrics for each ML model across the different feature sets Spontaneous dialog.

Feature	ML Model	Train Accuracy	Test Accuracy	Sensitivity	Specificity	F1-Score	AUC	MCC
Acoustic	SVM	69.57	70.65	28.95	98.15	44	0.78	o.40
	**XGB**	**74.77**	**75.00**	**44.74**	**96.30**	**59.65**	**0.73**	**0.50**
	RF	99.06	70.65	47.36	87.03	57.14	0.72	0.38
	DT	95.33	59.78	55.26	62.96	53.16	0.60	0.18
	KNN	69.62	70.65	42.10	90.74	54.23	0.73	0.39
	MLP	72.89	72.83	41.10	90.44	56.14	0.77	0.45
MFCCs	SVM	75.72	78.85	67.50	84.38	70.13	0.82	0.55
	XGB	96.3	79.81	72.50	84.38	73.42	0.83	0.57
	RF	100	78.84	70.00	84.38	71.7	0.84	0.55
	DT	100	69.23	62.5	73.44	60.97	0.68	0.36
	**KNN**	**100**	**80.77**	**70.00**	**87.5**	**73.68**	**0.89**	**0.59**
	MLP	74.89	78.84	67.5	85.93	71.05	0.78	0.55
GTCCs	SVM	81.15	74.77	68.09	76.69	69.57	0.79	0.48
	XGB	100	81.08	74.47	85.94	76.92	0.88	0.61
	RF	100	75.68	68.08	81.25	70.33	0.85	0.49
	DT	100	69.34	59.57	76.56	62.22	0.68	0.36
	KNN	100	72.97	57.45	84.37	64.28	0.84	0.44
	**MLP**	**98.84**	**81.98**	**82.98**	**81.25**	**79.59**	**0.89**	**0.64**

Table S 11: Individuals PD and HC class values in features independent (Spontaneous dialog)

Feature Name	ML Model	Precision		Sensitivity/Recall		F1- Score	
		PD	HC	PD	HC	PD	HC
Acoustic	SVM	0.88	0.68	0.37	0.96	0.52	0.80
	XGB	0.71	0.72	0.53	0.85	0.61	0.78
	RF	0.75	0.71	0.47	0.89	0.58	0.79
	DT	0.50	0.65	0.53	0.63	0.51	0.64
	KNN	0.75	0.71	0.47	0.89	0.58	0.79
	MLP	0.70	0.75	0.61	0.81	0.65	0.78
MFCC	SVM	0.84	0.82	0.68	0.92	0.75	0.87
	XGB	0.74	0.82	0.70	0.84	0.72	0.83
	RF	0.76	0.82	0.70	0.86	0.73	0.84
	DT	0.63	0.81	0.72	0.73	0.67	0.77
	KNN	0.78	0.82	0.70	0.88	0.74	0.85
	MLP	0.85	0.83	0.70	0.92	0.77	0.87
GTCC	SVM	0.81	0.81	0.72	0.88	0.76	0.84
	XGB	0.75	0.83	0.77	0.81	0.76	0.82
	RF	0.73	0.81	0.74	0.80	0.74	0.80
	DT	0.62	0.75	0.68	0.69	0.65	0.72
	KNN	0.76	0.77	0.66	0.84	0.70	0.81
	MLP	0.80	0.87	0.83	0.84	0.81	0.86

Table S 12: ML models performance with combination of two sets of features (Spontaneous dialog)

Feature	ML Model	Train Accuracy	Test Accuracy	Sensitivity	Specificity	F1-Score	AUC	MCC
Acoustics + MFCCs	SVM	87.80	79.55	62.22	97.67	75.68	0.90	0.64
	**XGB**	**96.59**	**80.68**	**71.11**	**90.70**	**79.01**	**0.90**	**0.63**
	RF	100	79.54	66.67	93.02	76.92	0.92	0.62
	DT	100	67.04	60.00	74.42	65.06	0.67	0.34
	KNN	84.88	73.86	53.33	95.34	67.61	0.82	0.53
	MLP	79.51	72.72	64.44	81.39	70.73	0.82	0.46
Acoustics + GTCCs	SVM	92.20	76.14	59.46	88.24	67.69	0.82	0.51
	XGB	90.24	75.00	54.05	90.20	64.52	0.88	0.48
	RF	100	72.73	59.46	82.35	64.70	0.86	0.43
	DT	100	62.50	64.86	60.78	59.26	0.63	0.25
	KNN	100	69.31	56.75	78.43	60.87	0.79	0.36
	**MLP**	**90.73**	**77.27**	**72.97**	**80.39**	**72.97**	**0.81**	**0.53**
MFCCs + GTCCs	SVM	81.89	78.85	58.54	92.06	68.57	0.85	0.55
	**XGB**	**100**	**81.73**	**70.73**	**88.89**	**75.32**	**0.89**	**0.61**
	RF	100	79.80	63.41	90.47	71.23	0.88	0.57
	DT	100	66.34	58.54	71.43	57.83	0.65	0.29

	KNN	88.06	79.80	82.93	77.77	76.40	0.88	0.59
	MLP	78.60	77.88	65.85	85.71	70.13	0.86	0.53

Table S 13: Individuals PD and HC class values in features combined (Spontaneous dialog)

Feature Name	ML Model	Precision		Sensitivity/Recall		F1- Score	
		PD	HC	PD	HC	PD	HC
Acoustics + MFCCs	SVM	0.97	0.71	0.62	0.98	0.76	0.82
	XGB	0.85	0.76	0.73	0.86	0.79	0.80
	RF	0.94	0.73	0.67	0.95	0.83	0.78
	DT	0.81	0.78	0.78	0.81	0.80	0.80
	KNN	0.90	0.70	0.62	0.93	0.74	0.80
	MLP	0.86	0.72	0.67	0.88	0.75	0.79
Acoustics + GTCCs	SVM	0.79	0.75	0.59	0.88	0.68	0.81
	XGB	0.77	0.76	0.62	0.86	0.69	0.81
	RF	0.84	0.75	0.59	0.92	0.68	0.82
	DT	0.78	0.84	0.78	0.84	0.78	0.84
	KNN	0.69	0.73	0.59	0.80	0.64	0.77
	MLP	0.77	0.81	0.73	0.84	0.75	0.83
MFCCs + GTCCs	SVM	0.81	0.82	0.71	0.89	0.75	0.85
	XGB	0.76	0.82	0.71	0.86	0.73	0.84
	RF	0.74	0.80	0.68	0.84	0.71	0.82
	DT	0.63	0.79	0.71	0.73	0.67	0.76
	KNN	0.81	0.84	0.73	0.89	0.77	0.86
	MLP	0.74	0.85	0.78	0.73	0.76	0.84

Table S 14: ML models performance for PD identification from speech with Combine Feature sets (Spontaneous dialog)

Feature	ML Model	Train Accuracy	Test Accuracy	Sensitivity	Specificity	F1-Score	AUC	MCC
Acoustic +MFCCs +GTCCs	SVM	86.45	81.52	65.79	92.59	74.63	0.87	0.62
	**XGB**	**100**	**83.70**	**76.32**	**88.89**	**79.45**	**0.88**	**0.66**
	RF	100	80.44	71.05	87.04	75.00	0.85	0.59
	DT	100	61.95	55.26	66.67	54.55	0.61	0.22
	KNN	84.11	78.26	60.52	90.74	69.69	0.87	0.55
	MLP	85.04	73.91	63.15	81.48	66.67	0.84	0.45

Table S 15: Individuals PD and HC class values in all features combined (Spontaneous dialog)

Feature Name	ML Model	Precision	Sensitivity/Recall	F1- Score

		PD	HC	PD	HC	PD	HC
Acoustic +MFCCs +GTCCs	SVM	0.89	0.80	0.66	0.94	0.76	0.86
	XGB	0.78	0.84	0.76	0.85	0.77	0.84
	RF	0.74	0.79	0.68	0.83	0.71	0.81
	DT	0.54	0.67	0.53	0.69	0.53	0.68
	KNN	0.82	0.81	0.71	0.89	0.76	0.85
	MLP	0.83	0.84	0.76	0.89	0.79	0.86

Table S 16: Features comparisons paired with t-test score of ml model (Spontaneous dialog)

ML Model / P - value	Acoustic vs MFCCs	Acoustic vs GTCCs	MFCCs vs GTCCs	MFCCs vs MFCCs+ GTCCs	MFCCs vs MFCCs +Acoustic	MFCCs vs GTCCs+ Acoustic	MFCCs + Acoustic vs ALL	GTCCs + Acoustic vs ALL	GTCCs + MFCCs vs ALL
SVM	0.0000	0.0000	0.5325	0.2760	0.1659	0.3181	0.0182	0.1319	1.0000
XGB	0.0018	0.0004	0.7322	0.2575	0.3466	0.2486	0.0497	1.0000	1.0000
RF	0.0015	0.0019	0.6024	0.4922	0.2014	0.0207	0.5136	0.0453	0.0576
DT	0.0429	0.3278	0.2586	0.5173	0.2505	0.7686	0.6318	0.4804	0.3874
KNN	0.0003	0.3666	0.0014	0.1972	0.1619	0.4120	0.1339	1.0000	0.0592
MLP	0.0000	0.0000	0.4337	0.0453	0.8088	0.8088	1.0000	0.1089	0.0065
